# Structural-demographic analysis of the Qing Dynasty (1644–1912) collapse in China

**DOI:** 10.1371/journal.pone.0289748

**Published:** 2023-08-18

**Authors:** Georg Orlandi, Daniel Hoyer, Hongjun Zhao, James S. Bennett, Majid Benam, Kathryn Kohn, Peter Turchin

**Affiliations:** 1 School of Letters, Osaka University, Toyonaka, Japan; 2 Italian School of East Asian Studies, Kyoto, Japan; 3 Seshat: Global History Databank, Evolution Institute, San Antonio, Florida, United States of America; 4 George Brown College, Toronto, Canada; 5 Evolution Institute, San Antonio, Florida, United States of America; 6 School of Business and Finance, Shanghai Normal University, Economics, Shanghai, People’s Republic of China; 7 University of Washington, Seattle, Washington, DC, United States of America; 8 Complexity Science Hub Vienna, Austria; 9 Federation of American Scientists, Washington, DC, United States of America; 10 Department of Ecology and Evolutionary Biology, University of Connecticut, Storrs, CT, United States of America; 11 Centre for the Study of Social Cohesion, School of Anthropology and Museum Ethnography, University of Oxford, Oxford, United Kingdom; University of Agriculture Faisalabad, PAKISTAN

## Abstract

This paper analyzes the collapse of the Qing dynasty (1644–1912) through the lens of the Structural Demographic Theory (SDT), a general framework for understanding the drivers of socio-political instability in state-level societies. Although a number of competing ideas for the collapse have been proposed, none provide a comprehensive explanation that incorporates the interaction of all the multiple drivers involved. We argue that the four-fold population explosion peaking in the 19th century, the growing competition for a stagnant number of elite positions, and increasing state fiscal stress combined to produce an increasingly disgruntled populace and elite, leading to significant internal rebellions. We find that while neither the ecological disasters nor the foreign incursions during the 19th century were sufficient on their own to bring down the Qing, when coupled with the rising internal socio-political stresses, they produced a rapid succession of triggering events that culminated in the Qing collapse.

## Introduction

In 1820, China’s economy accounted for 32.9% of the World’s GDP, by far the largest on earth [1]. Two hundred years later China has again claimed the distinction as the world’s largest economy (measured in terms of purchasing power parity; PPP) [2]. Between these two periods of productivity, China experienced a tumultuous period, typically dated from 1839 to 1949, which the Chinese still refer to as the “Century of Humiliation” (*bainian guochi*). After 1820, China’s share of world GDP began to shrink, and by 1870 it was less than half of that of Western Europe. The country suffered a series of defeats by external enemies and an extended sequence of droughts, famines, epidemics, internal strife, and rebellions. The Taiping Rebellion (1851–64), in particular, is the bloodiest civil war in recorded human history [3–5] in which over 20 million people died. China, under the Qing Dynasty during this period, became known as the “Sick Man of East Asia” (*Dongya bingfu*), ultimately ending in the Dynasty’s collapse in 1911.

Scholars working in different fields have proposed a panoply of explanations for the social and political breakdown of China’s last imperial dynasty [6]. Most of these theories, especially Eurocentric ones that explained China’s breakdown by invoking deep persistent cultural differences between China and Europe [7,8], have not held up well to further scrutiny [9–16]. Indeed, China’s dramatic economic rise after Deng Xiaoping’s reform and opening-up (*gaige kaifang*) has belied traditional views about China’s supposed ‘backwardness’, leading to a series of studies trying to explain the ‘Chinese miracle’ [17–21].

Others seek to place the collapse of the Qing within China’s longer imperial history, stretching back to the Han Empire of the late first millennium BCE [22–25]. This perspective sees the rise and fall of the Qing as just one in a long cycle that saw dynasties come and go every few centuries. Scholars have proposed a variety of models attempting to explain the dynastic collapses that have recurred throughout China’s imperial history. Usher [26] for example focused on how population growth stresses the distribution of material goods between farmers, rulers, and bandits, leading to unrest and eventually state collapse. Chen [27] as well as Chu and Lee [28] modeled the effect of such disruptive shocks as climate change and internal war (e.g., peasant revolts) on Qing state capacity. However, Zhao et al. [29] argue that climate shocks did not significantly impact the probability of revolts during the Qing. Instead, they show that the frequency of social revolts was largely prompted by financial volatility (itself driven mainly by periodic disruptions to the silver inflows the Qin required for their fiscal efforts) affecting price levels and, thus, hampering state capacity during shortfalls. In short, previous research has identified a number of competing mechanisms to explain the Qing collapse, yet many questions remain as to which of these struck the fatal blow or, if multiple forces were involved, how exactly did they interact.

An alternative perspective that has proven useful in explaining other cases of social and political breakdown in complex societies is provided by Structural Demographic Theory (SDT). SDT is a general framework for understanding the drivers of socio-political instability in state-level societies, and has been applied to a variety of historical settings, from ancient Rome, the Age of Revolutions in the 19^th^ century Europe, as well as contemporary USA and Africa [30–34]. An SDT explanation for dynastic collapse and regeneration during the Ming-Qing transition and then the Qing collapse was outlined previously by Goldstone [32]. His study demonstrated how growing popular immiseration, elite overproduction, and strains on government finance weakened the Ming rulers and led to widespread discontent, notably among elites frustrated at their lack of access to the highest positions [32,35,36]. He also suggested that similar pressures were at the heart of the Qing collapse a few centuries later.

Here, we attempt to resolve lingering debates about how key forces combined to erode social cohesion and weaken state capacity during the final years of the Dynasty’s rule, eventually ending in a major revolution that transformed China’s political system. We test alternate hypotheses about the end of the Qing Dynasty against empirical time-series data, combining this analysis with historical narrative delving into the unique features of Qing rule. We find that, while a variety of factors contributed to increasing unrest over the course of the 19^th^ and early 20^th^ centuries and ultimately led to the downfall of Qing rule, SDT offers the most compelling framework and best fits the data available. We thus conclude that the end of imperial rule in China was driven largely by internal socio-political strain brought on by demographic pressure, conflict among the ruling classes, and the inability of state institutions to maintain function in the face of these mounting challenges.

This paper is organized as follows. First, we outline the key theories proposed by scholars to explain the end of Qing rule before providing a brief explanation of the general mechanisms for social breakdown and state collapse proposed by SDT. Then, following a short summary of the history of the Qing period, we present three sections that detail, in turn, the three main drivers of instability according to the theory: popular immiseration, elite overproduction/intra-elite conflict, and the state’s fiscal crisis and loss of legitimacy. In these sections we review empirical, quantitative evidence tracking changes in these factors over time as well as providing qualitative discussion of the key forces driving their movement. Next, we trace the dynamics of socio-political instability under the Qing, testing the various explanatory hypotheses against the available empirical evidence. This analysis shows that the forces identified by SDT reveal rising tensions leading to an outbreak of catastrophic rebellion and, ultimately, to dynastic collapse. We close with a brief comparison of the Qing collapse with the nearly contemporaneous collapse of the Romanov Dynasty in Russia, further highlighting the utility and applicability of SDT for tracing societal dynamics and explaining periods of socio-political collapse.

## Alternate explanations for the fall of the Qing

As noted above, a host of explanations have been offered by scholars to explain how China transitioned from one of the world’s major power to the ‘sick man of East Asia’ under Qing rule. These generally fall into two main categories, which we will review briefly in turn (see the SM for further details on how we constructed and gathered data on these different measures): those focusing on *exogenous factors*, which can be subdivided into arguments citing competition from foreign states and those that place the causal force on environmental changes and ecological shocks; and theories about *endogenous factors* that undermined Qing functioning and stability.

***Competition between states*** is a common explanation, focusing on the increasing strain put upon the Qing during the late 19^th^–early 20^th^ centuries by contemporary imperial powers, notably England, France, Germany, Russia, and Japan [37–40]. This competition led to numerous wars, detailed below, including the so-called Opium Wars fought with the British and a series of conflicts with the Japanese. These conflicts resulted in dramatic loss of life, the ceding of some territory, and large financial strain. Over time, these arguments hold, the Qing simply lost the capacity to maintain stability. Further, some scholars argue that frustration at the government’s failure to keep foreign influence outside of China led to internal unrest, including sparking the Republican revolution that finally toppled Qing rule [41,42]. Here, we measure the strength of this external competition as the number of wars fought with foreign states (**External War)**.

***Environmental forces*** have often been cited in recent years as driving societal unrest and state collapse, including under the late Qing. Several recent studies have drawn a direct link between adverse climate conditions, which generated recurrent ecological shocks–such as famines, plagues, and floods–and social unrest under the Qing [27,28,43]. Such theories hold that the disruption to food production, loss of life, and the expense of recovery efforts both made the Qing more susceptible to foreign interference and also drove internal strife among affected populations. It is notable, however, that some scholars expressly disagree with such assessments, arguing that while ecological stress may have contributed to unrest under the Qing (and other Chinese dynasties), they fail to provide convincing explanation as the *sole* or even *primary* cause of Dynastic collapse when other factors are considered [29,44]. To measure the influence of environmental forces, here we use the time-series data on the number and severity of droughts (**Drought**) and famines (**Famine**) as collected by the REACHES project [45].

Other scholars focus on the dynamics of Qing society itself [23,26,32,46]. Such ***endogenous forces*** include frustration and unrest among a growing segment of the population facing food shortages due to disruptions from violent conflicts, lack of available productive land, or ecological distress, as well as the dissatisfaction of wealthy and powerful citizens–the elite–unable to procure the high-status positions they seek. Other endogenous factors include economic strain, both that faced by the population as a whole from rising commodity prices (rice, wheat, other key goods) and the fiscal difficulty experienced by the state as silver imports dwindled, trade imbalances grew, and the rising cost of dealing with exogenous disruptions put enormous pressure on available resources. Here, we measure immiseration among the population by tracking the availability of agricultural land per-capita (though other measures are possible and conform to the same pattern; see SM for details), intra-elite conflict as the passing rates in examinations for top administrative degrees and the numbers of applicants to hold high office, and state fiscal strain as the budget surplus / deficit held by the Qing over time. SDT is also an endogenous theory, focusing on the dynamics of the strain that rising demographic pressure puts on social structures. SDT notably combines different endogenous forces highlighted by other theories, into a composite temporal measure, termed the political stress index (**PSI**; see below for further details). We focus here on this composite measure, which offers more explanatory power than any factor in isolation (we discuss this in the SM). To measure the actual instability experienced by the Qing we utilize data on the number of internal conflict events–civil wars, armed rebellions, and insurrections (**Internal War**)–as an indication of growing instability and loss of state capacity and cohesion.

In the following sections, we detail the fundamental components of SDT and describe how we developed these various measures in constructing a PSI time-series. We then deploy all of these measures in analyses to determine which type of theory holds the most explanatory power for understanding the end of Qing rule.

## Structural demographic theory

SDT was first proposed by Goldstone [47] and further developed by Turchin [48,49], Nefedov [50], Turchin and Nefedov [25], and Korotayev et al. [51,52]. SDT is a *structural* theory because it represents societies as interactive systems consisting of three main compartments: non-elite population (in the Qing period, primarily farmers, over 90% of the population), the elites (the population stratum from which bureaucrats were recruited), and the state (imperial court and the bureaucracy that managed the fiscal and legal systems, as well as the army). It is *demographic* because it traces the numbers and well-being of different segments of the population. SDT distinguishes between *structural pressures* on social instability, which develop slowly (on the time scale of years and decades) and fairly predictably, and *triggers*, which are much less predictable but act to release accumulated structural pressures in outbreaks of political violence. SDT tracks three specific measures of instability—population impoverishment (“immiseration”), intra-elite competition and conflict, and declining state capacity. Following previous SDT analyses [47,53], these three measures are termed Mass Mobilization Potential (MMP), Elite Mobilization Potential (EMP), and State Fiscal Distress (SFD), which are combined into an overall Political Stress Index (PSI) by multiplying them together:

PSI=MMP×EMP×SFD

Below we explain how we operationalized MMP, EMP, and SFD for the Qing in the sections devoted to each of these structural drivers for instability. We maintained the original formation of Goldstone [32] and used in other studies, where the Political Stress Index (PSI) was conceptualized as the product of the three key components above. The multiplicative formulation captures the argument expressed in the original theory that the combined stressors on sociopolitical instability interact and mutually amplify the overall stress.

In previous SDT studies, societies are seen as starting with an “integrative phase,” where an initial consolidation leads to a period of relative political stability and prosperity, as well as military success against external enemies, often resulting in territorial expansion. Such integrative periods, however, are never sustained indefinitely. Eventually, the pace of territorial and economic expansion slows down or even reverses. Yet, in most cases the population continues to grow, resulting in falling output per capita, especially when demographic pressure causes increasingly marginal land to come under the till, as is typical in pre-industrial agrarian societies. This dynamic signals the start of the “disintegrative phase,” during which social stability is gradually undermined by a combined action of several interconnected processes.

First, falling per capita output leads to reduced access to productive land, price inflation, falling real wages, urban migration, and increased frequency of food riots and wage protests indicating widespread *immiseration of the populace*. Immiseration can also increase the likelihood of a major ecological disaster, as malnutrition, overcrowding, and the migration (both within and between societies) aid the spread of disease and put pressure on food resources. While the structural conditions of immiseration develop slowly, a change of climate, such as a prolonged drought or several years of anomalous temperature, can serve as a trigger of a major famine or epidemic. As population well-being declines, the potential for popular discontent and uprisings increases; this pressure for instability is termed the *mass mobilization potential* (MMP).

Second, rapid expansion of the population and the gains from the associated economic growth promotes upwards social mobility, yet generally the number of prestigious posts remains relatively inelastic resulting in *elite overproduction*—an increased number of aspirants for the limited supply of elite positions. Elite overproduction in turn generates increased *intra-elite competition* as rival elites and elite aspirants vie for state rewards, such as prestigious administrative positions or lucrative titles. As a result, elites become riven by increasing rivalry and factionalism, again destabilizing the society. This pressure is called the *elite mobilization potential* (EMP).

Third, population growth puts increasing demands on state capacity to maintain administrative organization, fund public works, and expand military needed to defend the state from both external and internal enemies. Yet, attempts to increase revenues cannot offset the spiraling state expenses, particularly when these efforts are met with resistance from the elites and the general population. As state fiscal distress increases and its capacity wanes, state institutions often lose legitimacy in the eyes of the population as they cease to perform their intended functions, be it managing famines, rooting out administrative corruption, or prosecuting successful wars [32]. This pressure is the *state fiscal distress* (SFD).

As these three coupled dynamics progress, the society experiences rapidly rising socio-political pressures, which can be combined in a single indicator, PSI (see *Political Stress Index (PSI) and Observed Political Instability* for details). At the same time, declining state capacity and legitimacy makes it increasingly difficult for governments to respond to these events by adopting needed reforms and/or suppressing unrest with force. Eventually, intra-elite infighting, coupled with elite-mobilized popular uprisings result in the breakdown of central authority. The most frequent outcome is a revolution or civil war (or a combination of the two), although in some cases the governing elites manage to avoid major bloodshed by adopting a set of reforms that acts to reverse the drivers for instability. More generally, while the road to crisis tends to be fairly stereotypical, emergence from crisis and the start of the next integrative phase is much less determined, because the different structural pressures underlying the instability can be relieved in many ways: by successful reforms (in a minority of cases), by transformative revolution, by population decline (resulting from a major epidemic or prolonged internal war), or by external conquest [31].

## Brief overview of Qing dynamics

**Fig 1:**
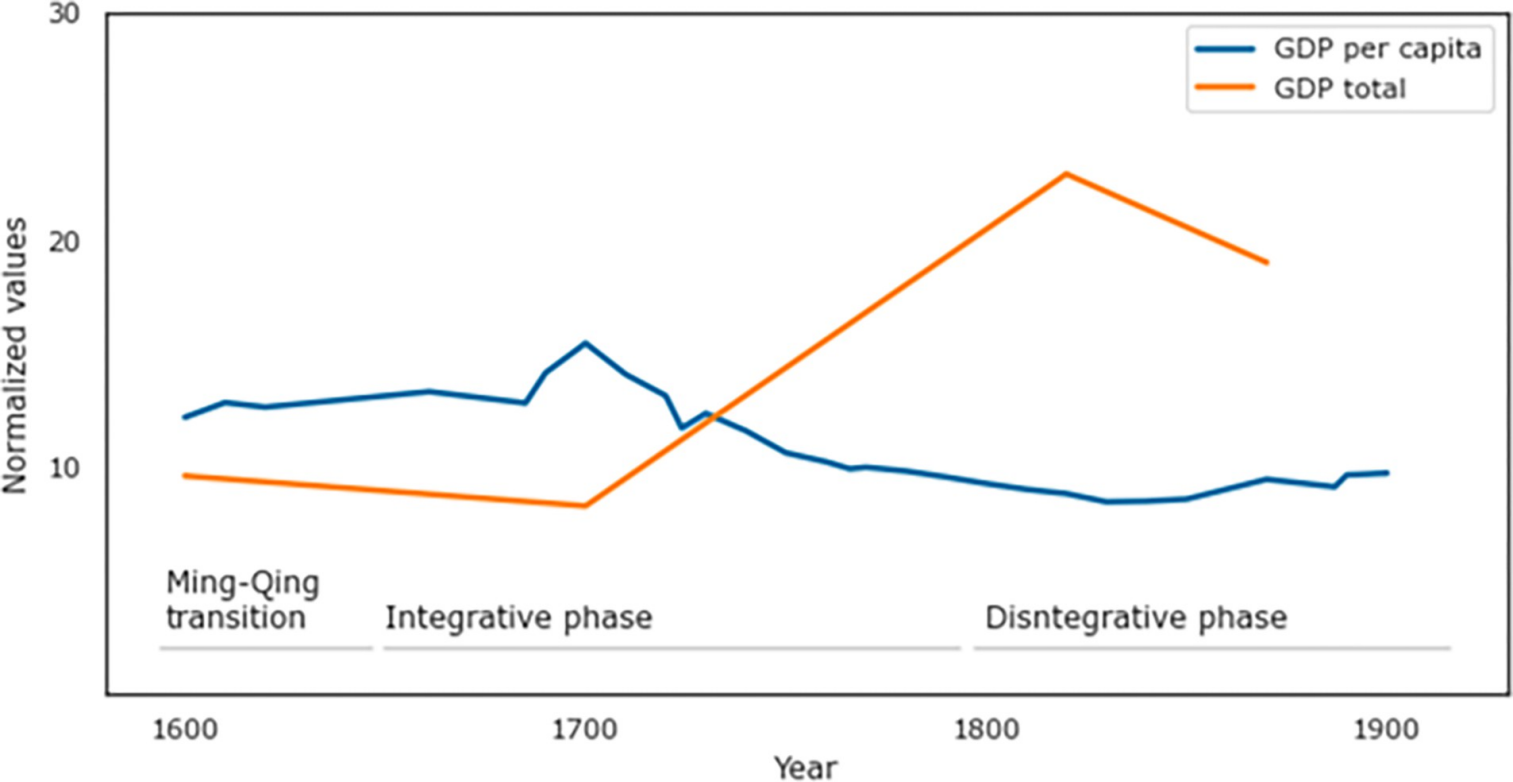
Economic growth and decline in China, 1600–1911. Total GDP in 10,000s of 2011 USD (orange) and GDP per capita in 100s of 2011 USD (blue). Our proposed dates of SDT phases for Qing rule marked as well. Source: *Clio-Infra* [56].

The Qing Dynasty was ushered in when the Ming Dynasty was overthrown by a rebellion led by Li Zicheng, which was in turn defeated by an external force, Jurchen-descendent Manchus, who captured Beijing in 1644. The Manchu Qing rulers suppressed several revolts by Ming loyalists during the early years of their reign, restored state power, and reshaped administrative systems. They took over the ideological mantle of rule from the Ming (the ‘Mandate of Heaven’) and oversaw a large, efficient administrative apparatus. They adapted some practices from the Ming and restored others present during earlier imperial phases, including a centralized hierarchy of councils and ministries overseeing a vast provincial system [54,55]. This consolidation of power enabled a period of rapid territorial expansion; at its height at the end of the 18^th^ century, the Qing claimed over double the territory as had the Ming [35,55]. The Qing also continued to promote economic growth, although it began earlier, during the Ming era, when China recovered from the bubonic Plague events of the 14^th^ and 15^th^ centuries. China’s participation in international markets expanded under the Qing, as foreign markets, particularly in Europe became increasingly hungry for Chinese produced goods such as silks and porcelain [35,37]. This led not just to increased overall output, but per-capita growth as well (Fig 1). In short, as is often seen with newly established dynasties, early rule under the Qing witnessed a clear expansion, or integrative phase in SDT terms, which lasted until the end of Emperor Qianlong’s reign in 1796.

As expected by SDT, the integrative phase did not last indefinitely, and was succeeded by a disintegrative phase. Already during the 18^th^ century, although total GDP continued to rise and Qing society remained fairly cohesive, population growth began outpacing productivity, causing per capita GDP to decline from its peak around 1700 (Fig 1). We also begin to see many of the usual signs of a society sliding into crisis (these are detailed in turn in the following sections). Notably, a number of rebellions plagued the second half of Qing rule, beginning with uprisings in Tibet and Taiwan in the mid-18^th^ century and increasing in frequency and intensity throughout the 19^th^ century. Recurrent droughts and famines put even more pressure on the already over-extended resource base required to support the massive population of Qing China. Additionally, the Qing faced increasing pressure from external states, particularly European imperial powers eager to acquire Chinese goods at low costs and to sell their own goods, including opium, into Chinese markets. Tensions rose between the Qing government and foreign merchants, leading to conflicts such as the Opium Wars in the 1840s–60s and resulting in concessions to the British, Japanese, and Russians as well as the French, Germans, and Americans. At the same time, the Qing’s declining economy and massive trade imbalance led to a shortage of available silver, causing deflation and weakening fiscal security. While deflation may have offered some relief to farmers and laborers, economic opportunities, particularly for the merchant classes, remained scarce driving up their discontent at China’s weakening international standing. This all made it increasingly difficult for the Qing to defend against British, French, and later German and Japanese incursions and limited their ability to suppress internal revolts. In all, the 19^th^ century witnessed a protracted disintegration of Qing rule, which we estimate below began just before the turn of that century, and ending with the Republican Revolution of 1911, which brought an end not only to the Qing Dynasty but to China’s imperial period [32].

In the sections below using the SDT lens we detail how growing popular immiseration, elite overproduction and competition, and declining fiscal security all combined to throw the Qing into this period of crisis. We argue that, while neither the ecological disasters nor the foreign incursions witnessed during the 19^th^ century were sufficient on their own to bring down the Qing, when coupled with the rising internal socio-political stresses, they produced a rapid succession of triggering events that culminated in the Qing collapse.

## Exploring the three drivers of SDT

### Popular immiseration

After a period of war and internal instability associated with the Ming-Qing transition during the 17^th^ century, China’s population began a rapid increase (Table 1). As a result of improved agricultural techniques and the introduction of new crops such as corn and sweet potatoes in the late Ming, increased acreage [57,58], and early industrialization, the Chinese population nearly quadrupled between 1700 and 1850, from 125 to over 400 million people [59–61]. Population growth was especially robust in the border provinces: Manchuria, Sichuan, Yunnan, and Guizhou.

**Table 1:** Population growth and declining per capita Arable Land in China. Sources [58,62].

Year	Total Population (mln)	Agricultural population (mln)	Arable land (mln mu)	Arable land per capita (mu)
1600	120	97	1,036	7.87
1766	200	170	1,036	4.98
1790	300	255	1,009	3.35
1812	350	297	1,050	2.89
1887	400	340	1,126	2.78

**Fig 2:**
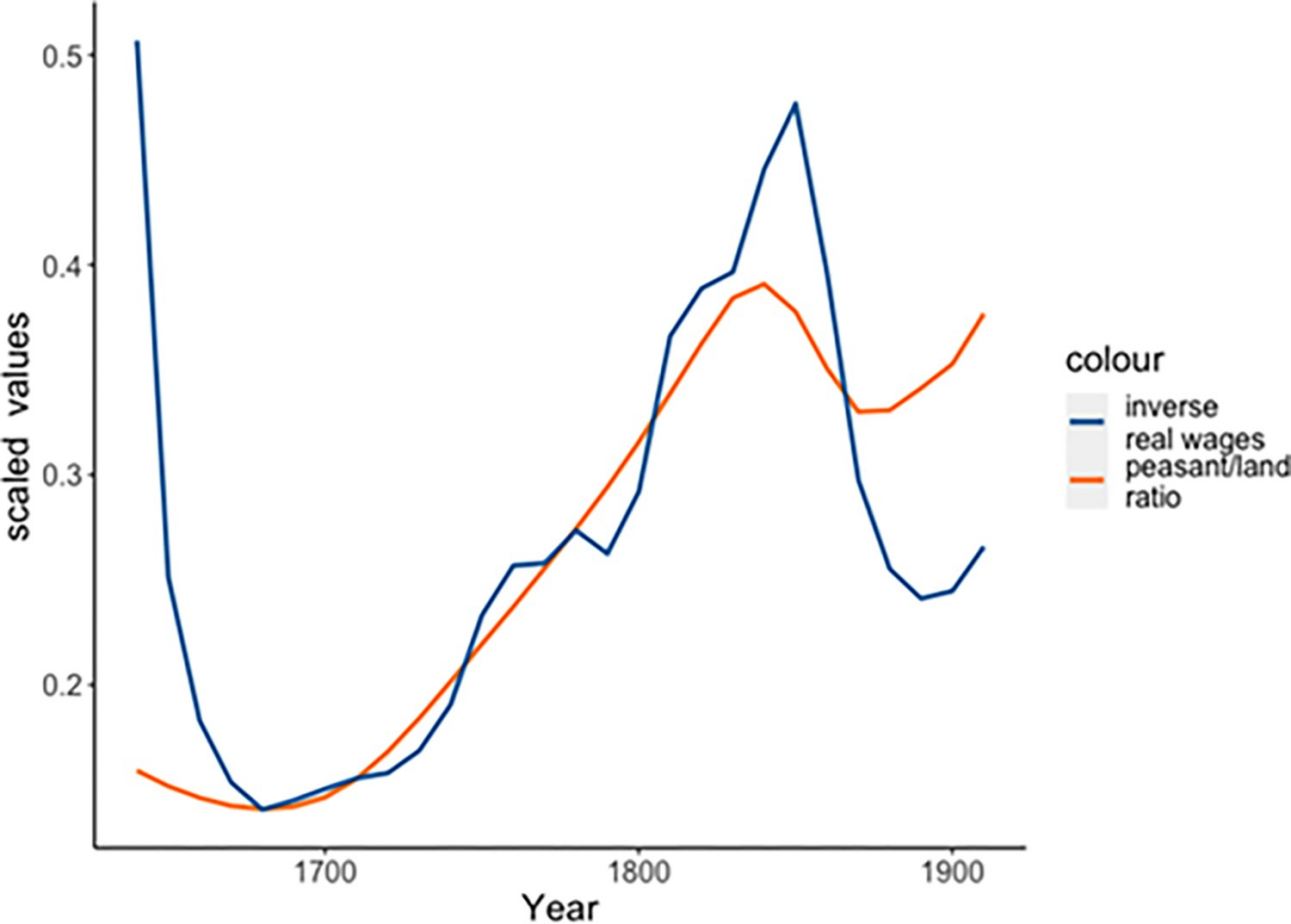
Availability of agricultural land per capita (orange) and inverse estimated real wages (blue), 1644–1912. Sources: Population estimates from the ClioInfra project; estimates of available arable land from [58,62]; real wage estimates after [63].

The first half of the Qing Dynasty was thus generally a period of economic prosperity as output–both agricultural and commercial–outpaced demographic growth. Estimates place GDP growth as nearly 30% from the onset of the Qing dynasty through the early 1800s [1]. Yet, over time this massive population growth resulted in declining arable land per capita, from 7.87 *mu* (a *mu* is equivalent to 614.4m^2^) in 1736 to a mere 2.78 *mu* in 1851 [58,62] (Table 1), while increasing numbers of labourers particularly in the growing cities along with periods of price inflation for staple goods led to falling real wages (Fig 2).

Another source of strain on the populace was taxation. Although taxation under the Qing was generally low compared to other parts of the world (see State Fiscal Health section for more details), as the increased size of the rural population led to a reduction of per capita output, many peasants increasingly struggled to meet their tax obligations. Moreover, regional diversity and the size of the Qing empire impeded the full monetization of taxes, and peasants were occasionally required to pay their taxes in rice, wheat, millet, or crops, despite falling productivity for these crops particularly in marginal regions [64]. In addition, during periods of silver scarcity, peasants were allowed to pay land taxes in copper, but the conversion (‘commutation’) rates were set by local magistrates with little oversight [65–67]. This allowed for corruption, as magistrates at times could manipulate the commutation rates to impose greater burdens on the peasantry while also withholding a considerable amount of tax income from state coffers [65,66,68]. There were also regional differences in the extent of such corruption, as the Qing government was able to retain more taxes in regions closer to Beijing, where the court could monitor the tax officials better [69,70], whereas in those places where state control was limited the phenomenon of over-collection (*fu shou*) by tax collectors who kept the lion’s share for themselves was more endemic [65].

Further, poor and unpredictable climatic conditions led to a series of food crises during the later Qing period as well, exacerbating the challenges of feeding a still growing population (see the *Exogenous Factors* section below for further details). While the Qing government built a nationwide system of public granaries to administer famine-relief systems and mitigate the impacts of these famines [71–74], support for these efforts was not consistent and over time proved ineffective at providing significant relief. An illustrative example of this issue occurred in 1792, when Emperor Qianlong found that Zhili’s reported grain reserve was grossly inflated and local magistrates had no capacity to fulfil his order to provide relief [64,75]. Corruption among local officials also impacted the frequency and size of the Qing state’s granary operations [70], and after the 1780s these granaries became less and less available [71]. Interestingly, despite these disruptions in productivity, rice prices (a principal staple food at this time) remained fairly stable through the first parts of Qing rule.

These shocks led to a large number of deaths, as did the numerous armed conflicts against both internal and external foes the Qing engaged in during the period, though overall the Qing population continued to grow. This put increasing strain on the Qing’s resources and management systems, leading to rising popular immiseration as we illustrate here.

Anthropometric data confirm these trends. Average stature, a useful proxy for biological well-being, declined during the late Qing China. Using prison records from the Australian Colony of Victoria, for instance, Morgan [76] estimates a decline in the height of unskilled Chinese laborers from 164 cm in 1810–50 to below 162 cm at the end of the nineteenth century. Baten et al.’s [77] quantitative study confirms that Chinese height stagnated and declined from 1850 onwards, underperforming even within the East Asian context. Other data point to a decline in household formation, marriages, and births by the late 19^th^ century, suggesting a society under substantial demographic stress [78,79]. This again is consistent with SDT, where a period of extensive demographic expansion is often followed by one of contraction as structural pressures put great strain on populations, leading to higher rates of death and lower rates of birth.

As most of the Chinese non-elite population (roughly 85%) were peasants, we focus on how, in spite of increased acreage brought into cultivation, the massive population growth during the late 17^th^ through early 19^th^ centuries resulted in decreased land per capita. Accordingly, we operationalize the Mass Mobilization Potential (MMP) for Qing China as an inverse of land/capita. As demonstrated above, the sharp decline in this ratio experienced during the course of the Qing is strongly associated with various signs of rising popular immiseration. We do see some signs of recovery at the end of the 19^th^ century, primarily due to population decline (Fig 2) [80,81]. We discuss the implications of this development below, but it appears that this relief came too late to save the dynasty form the various pressures that had built up by that point.

### Elite overproduction and intra-elite conflict

The elite in imperial China was mostly composed of scholar officials and degree holders [82,83]. There were also gentry—locally dominant landholders—and smaller landlords who held no degree, but the most powerful group of elites was composed by imperial scholars and degree holders. The military was thoroughly subordinated to the administrative elites, except towards the end of the nineteenth century (laying the foundation for a period characterized by the proliferation of warlords and military factionalism).

The fundamental force driving elite overproduction in the late Qing was the overall population increase. Not only did it foster popular immiseration, it also resulted in an increase in the number of aspirants for elite positions in government, whose supply overall did not grow (although there were fluctuations around this level). The combination of a static number of power positions and growing numbers of elite aspirants resulted in an explosion in the numbers of frustrated elites, many of whom became counter-elites, as was the case of the Taiping Rebellion’s leaders.

During the Qing period, top administrators were mostly recruited through the civil examination system (*keju*), which aimed to select candidates for the state bureaucracy. The examination system consisted of various levels of examinations and degrees:

- The most basic degree at the lowest level was the licentiate (*shengyuan*).
- The next level was the imperial student (*jiansheng*), who had passed the triennial qualifyingexamination (although this degree was more often than not purchased)
- Next came the *juren* who had passed the triennial provincial examination.
- The *gongshi* had to pass the metropolitan examinations
- At the top of the hierarchy were the *jinshi*, who had passed the palace examination [82–84].

Other examinations existed during the Qing dynasty, such as the ‘military examination’ (that had lower prestige than civil examinations) and the ‘translation examination’ (*fanyi kaoshi*). An alternative route to becoming a member of the elite was through the purchase of an academic title, bypassing the rigorous exams [82,84].

The problem of elite overproduction worsened during the 18^th^ century. During the early Qing, in 1691, 2500 aspirants participated in the Metropolitan examination, with 6.4% succeeding [83] (Table 2). The number of aspirants rapidly grew during the 18^th^ century, reaching nearly 6,000. At first, the passing ratio was kept relatively high by a temporary increase in the number of positions, but by 1850, on the eve of the Taiping Rebellion, the number of positions declined (Table 2). As a result, the passing ratio in 1850 became only 3.5% (it improved towards the end of the 19^th^ century, but, as we argue below, this was too late to save the dynasty).

**Table 2:** Increasing competition among *gongshi* to pass metropolitan examinations, 1691–1890. Sources [82,84,86].

Year	No. of candidates	Degrees awarded	Passing Ratio %
1691	2,500	154	6.2
1737	5,000	321	6.4
1742	5,993	310	5.2
1850	6,000	209	3.5
1890	6,000	328	5.5

Similar developments affected the provincial examination, especially in places such as Beijing, Shandong, and Sichuan. In the Beijing provincial examination of 1654, 6,000 candidates competed for 276 degrees, with a passing ratio of 4.6. The number of candidates spiked to 10,000 in 1748 and 13,000 in 1874, with the passing ratio collapsing to 2.3 and 1.8, respectively. In Shandong and Sichuan, the number of candidates tripled between mid-eighteenth and late nineteenth century, while the number of degrees awarded did not increase [82,84].

An additional factor was a sharp increase in the number of expectant officials—those who were qualified but for whom no position was available [32,82,84]. Reduced quotas, bribes, and scandals of various types triggered major riots in 1657, 1711, 1723, 1741, and 1750 [82], indicating growing discontent with the system. Things became worse in the late 18^th^ century, when the Qing decreased the fixed quotas for *jinshi* (the top degree), limiting the number of open positions.

Although we lack exact figures, it is likely that the number of elite aspirants increased not only because of the larger source population, but also because there was a substantial growth in the wealthy merchant class, which was the primary source of new aspirants aiming to join the ranks of the literati (see *Discussion* for more details). The number of provinces and counties did not increase, however, meaning that the number of elite positions stayed roughly constant (after an initial increase in the early 18^th^ century) [82,85,86]. Notably, the number of awarded *jinshi* degrees, the top palace exam, constantly decreased, touching its lowest point in 1796 when the number of admissions per subject had been a mere 81, the lowest ever recorded [87]. Indeed, the number of *jinshi* holders under the Qing [26,88] was not much higher than what was awarded during the Ming dynasty [84,88], despite the fact that the Qing population was four times that during the Ming. As a result, by the later Qing period there was a sizeable population of wealthy families, who could afford the cost of formal education, but whose hopes of placing a son in a top administrative position were frustrated.

The lowest-level degree, the *shengyuan*, also became a source of conflict in this period. As competition for the higher degrees intensified due to the declining passing ratio, the numbers of *shengyuan* who could not hope for higher office kept growing. This situation was exacerbated by the Qing policy allowing the purchase of degrees. Between 1764 and 1871, the number of officials decreased slightly, while the proportion of officials who attained their positions by exam dropped by one-quarter, while those who purchased degrees increased from less than a quarter to nearly half of all holders (Table 3). Although official academies were mushrooming across the empire, the examination system and the formal bureaucracy remained almost unchanged [32,82,84]. In this competitive environment, resourceful literati ventured into semi-legal or illegal enterprises. Some of them included the widespread though formally illegal tax commissioner (*baolan*), or the ‘pettifogger’ (*songshi* or *songgun*), a form of often corrupt attorney [88]. However, the Qing law prohibited litigation at the county offices on behalf of others, and the pettifoggers were granted no legal status [88].

**Table 3:** Percentage of Qing officials gaining degrees through civil examination (all levels), Yin privilege, and purchase. ‘Yin Privilege’ refers to the hereditary privilege enjoyed by the sons of higher officials who could inherit the title without standing for examinations. Sources [82,84,86].

Year	No. of Officials	Examination	Yin privilege	Purchase	Other
1764	2,071	72.95%	1.1%	22.4%	4.0%
1840	1,949	65.70%	1.0%	29.3%	4.0%
1871	1,790	43.80%	0.8%	51.2%	4.2%
1895	1,975	47.90%	1.2%	49.4%	1.5%

In addition, the inelastic civil examination system often caused discontent among failed literati, who could become hostile towards it. Over the years they spent studying for a degree they would never obtain, they had acquired the know-how necessary to organize and direct a political uprising. Indeed, almost all the highest-ranked leaders of the Taiping Rebellion had failed the civil examinations multiple times. The uprising was commanded by Hong Xiuquan, an ethnic Hakka who failed the civil examinations four times [5]. Hong Rengan, the cousin of Hong Xiuquan, had failed the examinations five times; and the same holds for many of the movement’s leaders [89,90]. The Taiping Rebellion is an extreme example of what can happen when frustrated aspiring elites have at their disposal the discontented masses, which they can mobilize for their intra-elite battles [3].

Further, there was a growing ethnic and cultural divide among top officials. The Qing established translation examinations for Manchus, Mongols, and other non-Han groups, which were aimed at promoting inter-ethnic equality, but resulted in giving advantage to these minority ethnic groups. The ethnic composition of the leadership in government positions before the Taiping Rebellion reflects a clear imbalance between Manchu and Han Chinese governors [91,92]. During the first period, the Qing government relied much more on Han Chinese Bannermen and Manchus. In 1668 governors-general in the Shaanxi-Shanxi area were all Manchus by edict [91]. Ethnic quotas were a source of inter-ethnic tension, since Han Chinese aspirant elites considered it unfair that a significant part of top positions were occupied by Manchus and Mongols, in spite of the fact that Han were by far the largest ethnic group of China. Manchus and Mongols also enjoyed a number of privileges, such as hereditary quotas and easier examinations, that greatly facilitated the passing of civil exams [82,84,93]. Evidence of such inter-ethnic tension is provided by the sharp decrease in the use of Manchus in provincial leadership after the Taiping Rebellion. Before the Taiping Rebellion the Manchus accounted for 35.9% of governor-general (*zongdu*) and 26.5% of provincial governor (*xunfu*); after it, both percentages had decreased respectively to 27% and 16.2% [91].

The intensification of competition resulting from the increasing numbers of elite aspirants, the decrease in the number of admissions at the civil examinations, the low success rate (1.6 percent) for the *shengyuan* taking the *jinshi* examination [82–84], the fixed quotas for Manchu and other groups [82,84] put an unbearable level of stress on the aspiring elites [82–84,94]. We quantify these social stresses, or elite mobilization potential (EMP), by focusing on one such dynamic: the number of *jinshi* degrees granted per capita. As the top degree, the *jinshi* were a major focus of aspirants’ attention, and as we saw above the various degrees tended to display similar trends. Tracking the number of *jinshi* degrees awarded per capita thus offers a viable measure of EMP.

### State fiscal health and legitimacy

During its earliest phase, 1644–1681, the Qing state had essentially no fiscal reserves due to the costs of consolidating its rule after the overthrow of the Ming. The Qing rulers were particularly sensitive towards anti-Manchu revolts, such as the Three Feudatories’ rebellion (1674–1681). Suppressing this revolt reportedly wiped out 85% of the surplus generated by the Board of Revenues, one of the six Ministries under the Department of State Affairs in Qing China, reducing it to as low as 3.32 million taels in 1678 [62,95,96]. This coincided with a period of economic depression, driven by a reduction in silver, the so-called Kangxi Depression (1660–90) [97,98]. Fortunately for the Qing, state capacity was being restored at this time and population was productive, so the coffers refilled rapidly.

**Fig 3:**
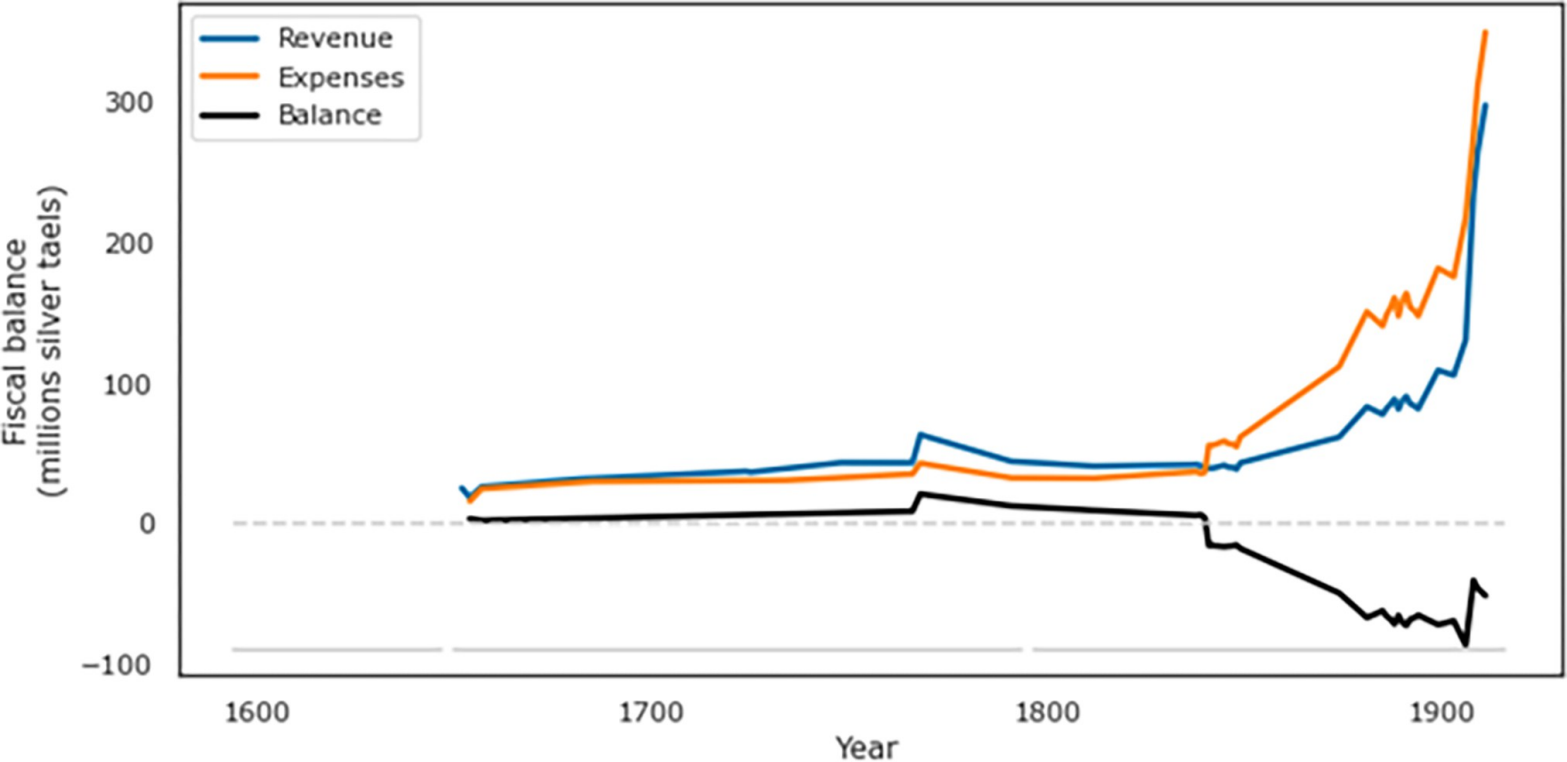
Fiscal balance under the Qing, 1652–1911, showing fiscal balance (black) calculated as estimated total revenue (blue) minus estimated total expenditures (orange). Sources [62,95,96,100–102].

After 1681 the Qing dynasty was able to generate a consistent surplus, building up a sizeable cash reserve, as revenues outpaced expenses (Fig 3). The most significant factor stressing the fiscal health of the Qing Empire (similar to other pre-modern states) was the need to pay for military campaigns against both external and internal enemies. However, fiscal security was maintained despite expensive Dzunghar campaigns in 1715–26 that cost around 50 million taels, the spending of an additional 9 million taels to suppress Zhu Yigui’s rebellion in Taiwan (1721), and the large military outlays in the suppression of the Tibetan tribes in Jinchuan (1771–76) [99]. During the eighteenth century expenses continued to mount, particularly the costs of suppressing internal unrest. After 1777, the Board’s cash reserves were nearly wiped out by campaigns against the White Lotus rebellion that cost around 53 million taels [95]. Another major campaign in Taiwan (1787–88) cost an additional 10 million taels [62,95], while the first and second Gurkha campaigns (1788–89, 1791–92) cost about the same amount again and the campaigns of annihilation of the “ocean bandits” led by Cai Qian (1802–10) cost another 7 million taels [62].

Despite these expensive conflicts, the Qing finances were in a relatively healthy state, with revenues exceeding outlays, until 1840 (Fig 3). This fiscal equilibrium was shattered during the nineteenth century, when the state outlays entered a period of accelerating growth (Fig 3). While administrative costs remained largely the same, other expenditures skyrocketed toward the end of the dynasty, due to the payment of war indemnities, major reforms, and modernization of the various state apparatuses. Notably, the Qing spent more than 290 million taels to suppress the Taiping Rebellion (1850–64), drawing in contributions from merchants needed to supplement the state reserves. In addition, the suppression of the Nian Rebellion (1853–68), the rebellion of the Hui people (1862–73), and many other small-scale revolts in the south-western provinces (1851–73) drained around 23.55 million taels per year [96,100,101]. Another 70–80 million taels were spent for the expedition into Xinjiang in 1875–1878, whereas around 30 million taels were spent to cover the costs of the Sino-French War (1884–85). Moreover, the creation of the Northern Fleet, and the payments of war indemnities, including those for the Opium Wars and the Boxer Rebellion, put the Qing fiscal health under significant stress, as the total amount of expenditures exceeded 900 million taels [101,102]. The Qing’s ability to finance these growing costs was additionally hampered by a lengthy period of financial depression, again due to shortcomings in the supply of silver coming into China as well as major outlays of silver to pay for the growing quantity of opium being imported into the country [103,104].

The Qing’s fiscal strain was exacerbated by their commitment to a light tax policy, which had been an axiom in Chinese history since the Han dynasty [105], and was continued by the Qing government [106]. Indeed, taxation in China was lighter than in other large contemporary polities, such as Tokugawa Japan or Western European countries, in part because the Qing often provided relief in the form of tax exemptions, tax freezes, and rent limitations [64,68]. While other tax sources increased during the late phase of the dynasty, land taxes remained the major source of government income until the late nineteenth century. Maritime taxes only increased significantly after 1885 and while other customs type dues increased and their revenue generally rose during the nineteenth century, combined these taxes were overshadowed by the increased military expenses. As such, while revenues increased, so did expenses and to a degree that still led to fiscal shortage. Rather than impose further taxes directly on producers, the Qing government sought to raise needed revenue by increasing the selling of degrees. Between 1820 and 1850 more than 315,000 degrees were sold [62,82]. Moreover, in the frenzy of selling degrees to raise funds for the pacification of rebellions, the Qing government de facto legitimized what had been hitherto considered corruption. The increase in degree-holders who struggled to find posts in the administration not only exacerbated tension among elites, but also eroded the legitimacy of the Qing regime. The state legitimacy was further undermined by a string of disastrous defeats against the external enemies.

The absence of a modern, central banking system during this period further contributed to the tight fiscal constraints faced by the Qing. The state was unable to ‘borrow’ or draw heavily on future debt to finance these expenses that continued to mount through the 19th century. It was only during the late Qing Dynasty that more modern-style banks emerged in China, influenced by Western banking systems. In 1905, the first true central bank, known as the *Da Qing hubu yinhang* (the Bank of the Board of Revenue of the Great Qing), was established. This proved too late to offer significant relief for the state’s fiscal woes, however. Indeed, only after the 1911 Xinhai Revolution and the subsequent formation of the Republic of China were comprehensive financial reforms were initiated to modernize the banking sector. We thus do not include any measure of banking activities or estimates of public debts in our analysis, as they played no major role in Qing fiscal life until after our period.

We operationalize State Fiscal Distress (SFD) by the fiscal deficit (difference between expenditures and revenues), scaled by revenues.

### Results: Testing alternate hypotheses about the fall of the Qing

To assess quantitatively the dynamics of instability throughout the Qing period, we constructed a time-series measure of internal conflicts, counting the frequency of conflicts by decade (see SM for details). This reveals the level of unrest experienced under the Qing over time, which we use as our response measure to test which body of theory best predicts these dynamics. We then explored how well our *endogenous* measure (PSI) explains this instability compared with the *exogenous* measures described above.

### Political Stress Index (PSI) and observed political instability

**Fig 4:**
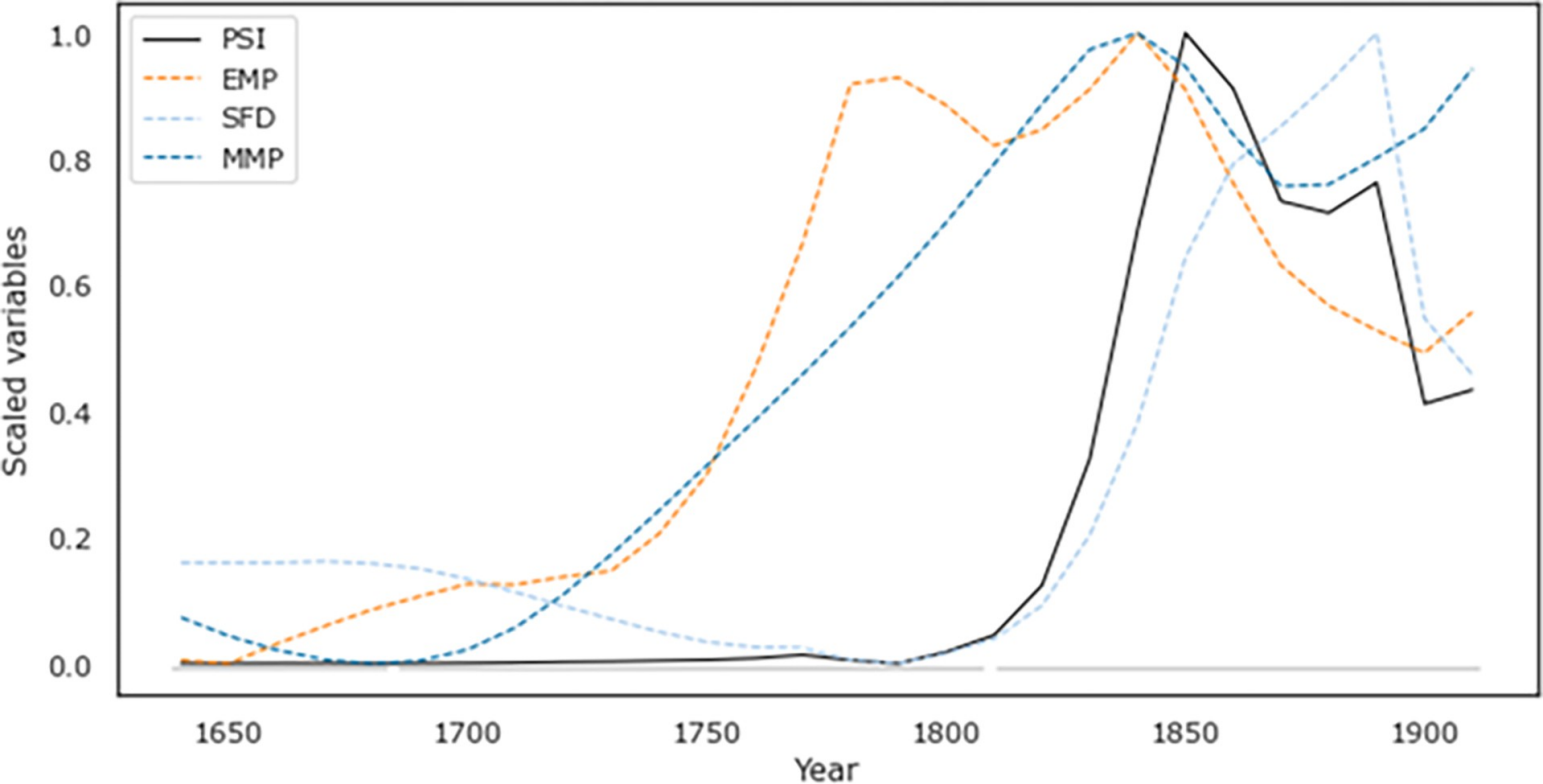
Political instability index PSI for the Qing dynasty showing the main components MMP, EMP, and SFD discussed in the text. Sources as detailed above.

SDT quantifies structural pressures for instability using the three indices, MMP, EMP, and SFD, which have been operationalized in the three preceding sections (*Popular Immiseration*, *Elite Overproduction and Conflict*, and *State Fiscal Health*; for calculation details, see the SM). When we plot these three indices against time, we observe that all three measures start at relatively low levels in the early Qing and then grow towards the end of the dynasty (Fig 4). The MMP and EMP curves rise throughout the eighteenth century and reach their peaks in the early nineteenth century. In contrast, SFD begins its ascent later, around 1800, and reaches its peak also later in the nineteenth century. Overall, PSI peaks in 1840–50, but its decline after that peak does not bring it back to the low levels characterizing the first half of the Qing Dynasty, suggesting that substantial pressures for instability persisted to the end of the dynasty.

According to SDT, PSI should be a leading indicator of observed instability. We assess the accuracy of this prediction by exploring evidence for internal unrest, including violent revolts and major outbreaks of civil conflict. After the Three Feudatories Revolt (1644–81), which was an “aftershock” of the Ming-Qing transition, China enjoyed a century-long period of internal peace and order, interrupted only by minor, localized revolts. The next major insurrection, which required a force of 300,000 soldiers to suppress, was Lin Shuangwen’s rebellion in Taiwan (1788) [62]. This was followed by a series of massive uprisings: The White Lotus (1794–1804), the Taiping (1850–64), the Nian (1851–68) rebellions, and the Dungan revolts (1862–77, 1895–96). The Dungan revolts were nearly as destructive as the Taiping Rebellion, causing a population loss of 21 million people, with Gansu and northern Xinjiang losing about three-quarters of their populations [107].

**Fig 5:**
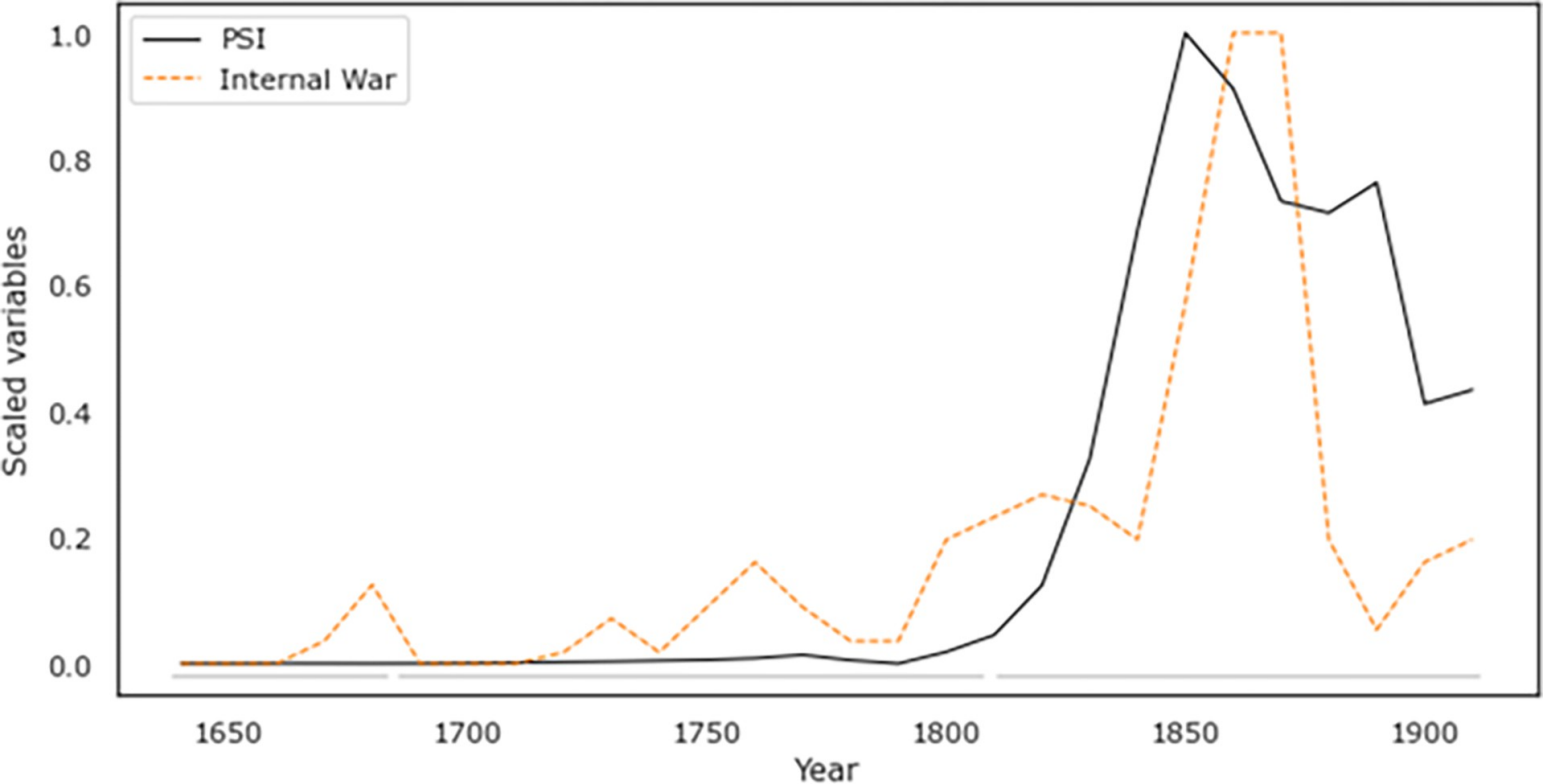
PSI compared with the relative number of internal wars during the Qing. For data sources and methodology, see SM.

Overall, our measure of observed instability under the Qing (Internal War) shows a similar dynamic as the PSI, remaining at relatively low levels during the first half of the dynasty before jumping sharply in the early 19^th^ century (Fig 5). SDT thus appears to be a good match, rising roughly in lock-step–though, critically, earlier–than the spike in internal war seen in the late 19^th^ century.

### Exogenous factors: Climate and external war

As discussed in the Introduction, a number of studies in recent years have argued that climate variability in temperature and precipitation, as well as shorter-term ecological shocks, famines and droughts, has had a significant, positive effect on the incidence of warfare, revolt, and dynastic change in China throughout the imperial period [108–115]. Other researchers [29,44,116], however, argue that, while climate shocks might have effectively amplified or accelerated decline, these factors were not the chief causes of crisis, because many instances of ecological shocks did not lead to major unrest. Zhao et al. [29] and Zhao [117,118], for instance, argue that human-induced financial factors such as major economic depressions and deflationary periods played a more important role than climate fluctuations in the fall of the Qing dynasty. Indeed, even those studies that argue otherwise acknowledge that other factors, such as population, were equally important factors affecting the agrarian economy of late Qing [119].

**Fig 6:**
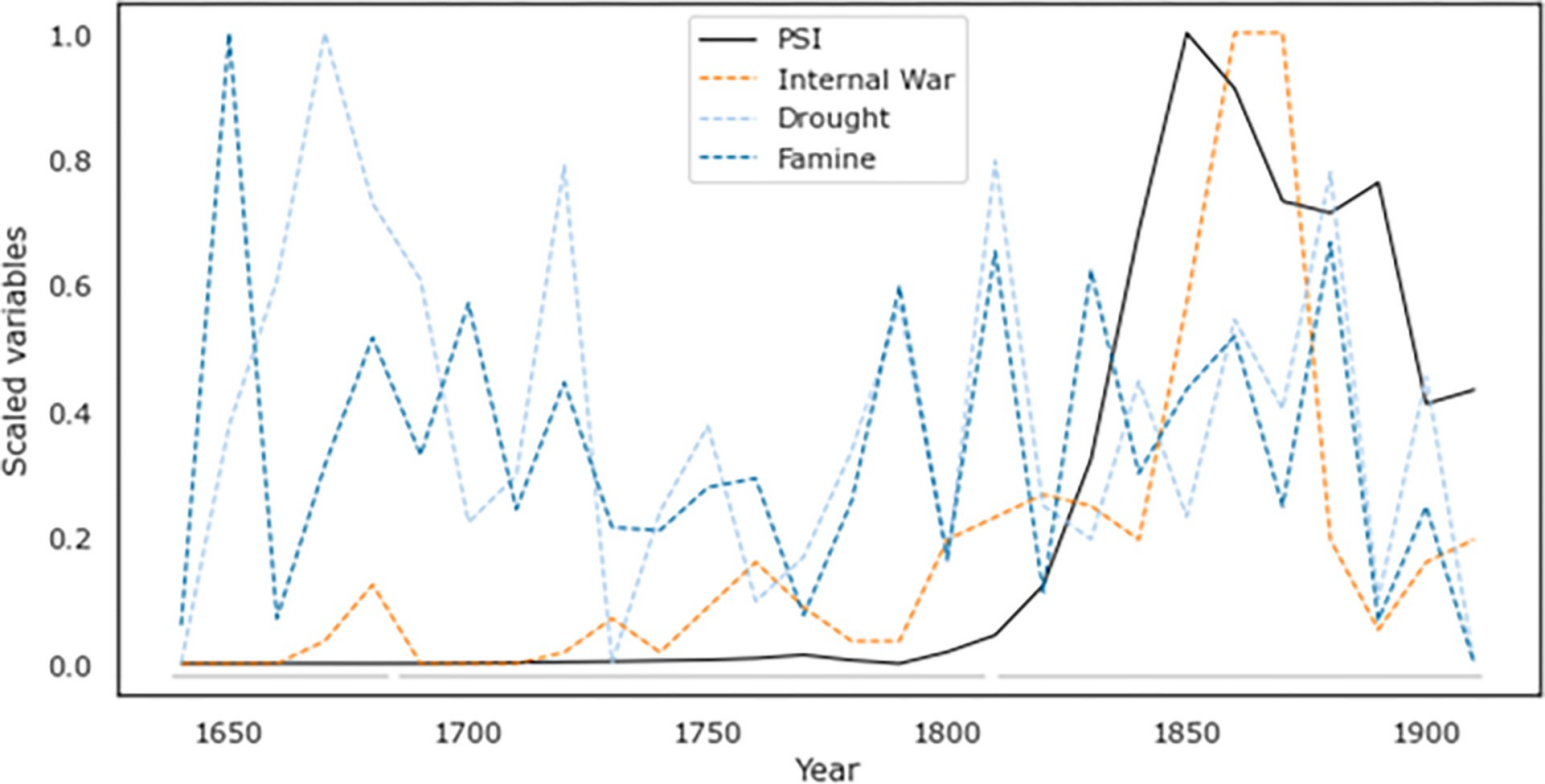
Major ecological shocks experienced under the Qing, 1644–1911. Source: REACHES climate database [45,121]. For data sources and methodology, see SM. Internal wars and our PSI measure shown for comparison.

We explored these contrasting claims by tracking major ecological shocks, focusing on famines and droughts, following the arguments made in previous scholarship (see Appendix for further details). Overall, we find that major outbreaks of drought and famine were fairly ubiquitous throughout our focal period and often co-occurred (Fig 6). As we see, following a major famine and drought in north-western China in 1630–31 which contributed to the collapse of the Ming Dynasty, there were no massive famines in China until the end of the 18^th^ century [120], though a major drought occurred in the 1720s and there were relatively minor episodes recurrent throughout. These disasters picked up pace and intensity in the 19^th^ century: two major famines occurred in 1846–49 and 1850–73, the latter thought to be a contributing cause of both the Nian and Taiping Rebellions. [120] Another famine in northern China in 1876–79, resulting in estimated 9–13 million deaths [120], followed by yet another famine in 1896–97 which perhaps helped spark the Boxer Rebellion [38].

Although there is a clear temporal link between some of these disaster events and major revolts, the Qing nevertheless weathered them. Social instability remained relatively low during the later 17^th^ and throughout the 18^th^ centuries despite the occurrence of significant ecological disasters (Fig 6). In these periods, the Qing were able to recover and maintain most state functions, limiting the impact on popular well-being by measures such as the public granaries mentioned above. Yet in the later part of the Dynasty (as PSI was on the rise), the Qing became less and less equipped to manage these events. This suggests that ecological shocks may have amplified crises, particularly by exacerbating popular immiseration in the late 18^th^ and most of 19^th^ centuries, but they do not appear by themselves to be a principal driver of the unrest and violence that peaked during the middle of the 19^th^ century.

**Fig 7:**
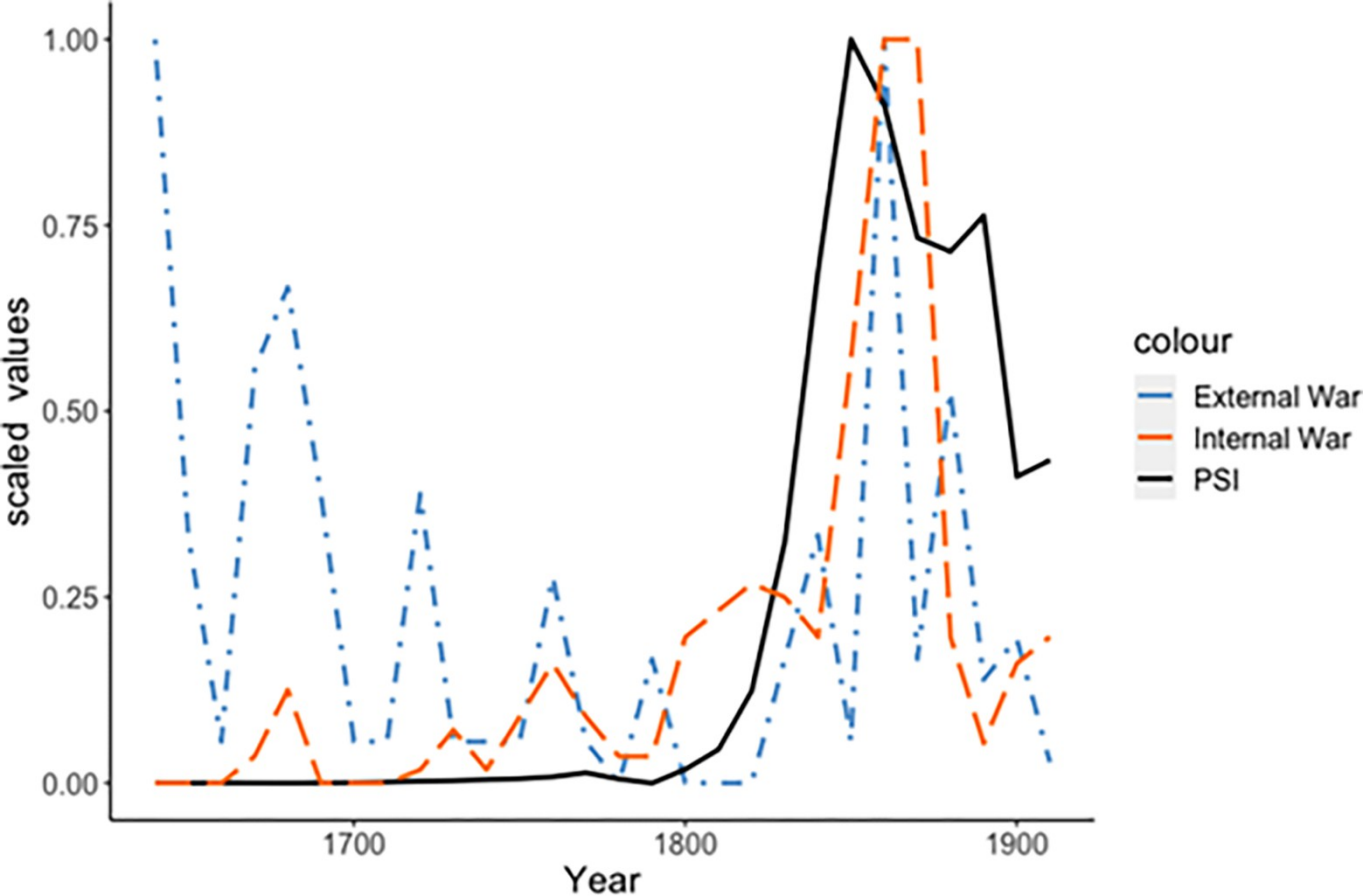
Relative number of external wars during the Qing Dynasty, 1644–1912. Sources: [95,96] Internal wars and our PSI measure shown for comparison.

The military history of the Qing Empire can be divided up in two periods, separated by a relatively peaceful period around 1800 (Fig 7). Most campaigns in the early period were successful, such as the Ten Great Campaigns (1755–92) which extended the empire or solidified Qing control over border territories. During the nineteenth century, however, as the Empire was increasingly weakened internally by structural-demographic pressures along with the strain from continuing ecological shocks, external wars became more destructive and politically destabilizing, resulting in loss of prestige, territorial concessions, and indemnities to pay. In 1842, Qing forces were easily defeated by the British army, and China was forced to accept the unequal terms of the Treaty of Nanking. In 1860, at the end of the Second Opium War (1856–60), China was forced to sign the Treaty of Tianjin. In 1858, seizing the opportunity, as the Qing was losing the Second Opium War, Russia annexed part of the Amur valley. Following the Mudan incident (1871), Japan annexed the Kingdom of Ryukyu, a tributary state of the Qing, and in 1874, it invaded Taiwan that was then controlled by the Qing government. In 1888 the British obtained Tibet’s renunciation of suzerainty over Sikkim [122]. In 1895, China was defeated in the First Sino-Japanese War (1894–95), and as a consequence of this outcome, it ceded Taiwan, the Pescadores and the Liaodong peninsula to Japan [123]. These geopolitical setbacks often triggered huge anti-government revolts. China’s loss in the two Opium Wars for instance gave impetus to the Self-Strengthening movement (*ziqiang yundong*, 1860–95), a period of radical institutional reforms aimed at modernizing the Qing’s industry and military. Protesting against the terms of the Treaty of Shimonoseki (1895), a number of notables led by Qiu Fengjia proclaimed the short-lived Republic of Formosa (23 May 1895–21 October 1895), the second-earliest republic of Asia, after the Lanfang Republic (1777–1884) [124].

External warfare then presents a similar picture to ecological shocks discussed above: On one hand, the 19^th^ century conflicts with foreign states track well with the rise in internal unrest and instability during this time, perhaps suggesting a causal relation; on the other hand, the high number of wars fought in the 17^th^ century do not correspond to a high degree of instability, and indeed the Qing survived these wars and retained imperial authority for over a century. Further, it is notable that the increase in external conflicts in the 19^th^ century either coincides with or *follows* the rise in internal unrest, unlike the PSI measure whose rise clearly precedes both.

Further, these campaigns against external foes were not entirely separated from internal pressures. Constant, and unsuccessful, warfare after 1830 was a major factor in driving up the SFD curve as detailed above (Fig 4, blue line) and, thus, contributed to rising overall PSI in this period. Additionally, the Opium Wars, the unequal treaties, and the defeat at the hands of former tributary states, such as Japan, eroded the Qing’s state legitimacy and fueled discontent among the population, as testified by a number of massive anti-Qing protests and movements. Anti-Qing sentiment finally came to a head in in 1911, when the Railway Protection Movement (*baolu yundong*) in Sichuan protested against the Qing’s plan to nationalize local railway projects and transfer control to foreign banks [41,42]. The suppression of the Railway Protection Movement gave impetus to the Wuchang uprising (1911), which in turn led to an ethnic clash in Lhasa that resulted in the end of the Qing rule in Tibet, whose control had already been eroded by the Treaty of Lhasa in 1904 and the Anglo-Chinese Convention in 1906 [125]. Anti-Manchu riots erupted also in Xinjiang and Mongolia, with the result that Outer Mongolia declared its independence from the Qing dynasty in December 1911. The Xinhai Revolution (1911–12) put an end to 276 years of Qing rule. As such, although the Qing government had to constantly cope with turmoil and sociopolitical unrest, the military defeats from mid-nineteenth century to the end of the dynasty contributed significantly to the eventual collapse of the dynasty in 1911.

### Quantitative test of relationship between hypothesized factors and internal war

The above results demonstrate a clear correspondence between the PSI curve and internal warfare curve, whereas the dynamics of the exogenous factors we explore here do not match well with instability. Critically, as noted, PSI rises *before* the major spike in instability, supporting the causal role of these endogenous, structural forces. Formal regression analysis confirms these results. When all of these factors are considered together, PSI stands out as displaying the highest correlation with the observed dynamics of Internal War. Finally, a lagged regression analysis likewise indicates that PSI is a *leading* indicator of observed instability; the best model (highest R^2^) for predicting InternalWar is the level of PSI reached one decade before. The ecological factors and external conflicts do not display significant relationship with internal warfare, either in synchronous or lagged regression.

Exploring the different factors that make up our PSI measure–MMP, EMP, and SFD–separately produce weaker results than PSI. Thus, the combination of the three primary forces highlighted by SDT display a stronger relationship with internal conflict than any in isolation, further supporting our conclusion that SDT offers the most compelling theoretical explanation for the decline and fall of the Qing dynasty.

### Response measure: Internal war

Following the procedure developed in Turchin and Nefedov [25], we defined “instability-years” as all years during the Qing period that there was an internal war, such as rebellion or insurrection. For example, a rebellion that lasted from 1850 to 1852, gave as 1850, 1851, and 1852 instability-years. If there was another rebellion at the same time elsewhere, it would add more instability-years (thus, 1852 would be counted as many times as there were concurrent rebellions in that year). Next, for each decade between 1640 and 1910, we added together all instability-years for an overall index of political instability during the decade. This procedure, admittedly, generates a fairly crude index of instability, because it treats all internal wars as same in magnitude. On the other hand, more intense conflicts tend also to last longer, and this aspect is captured by the approach (longer conflicts generate more instability-years). Overall, it provides us with a semi-quantitative measure that can be calculated for those historical periods, for which the estimates of the numbers of people killed are unreliable, or even absent.

List of conflicts involving the Qing were adopted from data provided by the Correlates of War [126,127] project as well as the Conflict Catalogue [128], with additional information taken from the *China War Chronology in Various Dynasties* volume [129]. We record from these sources 57 individual internal conflicts, along with 55 external conflicts used to calculate the **External Warfare** factor (detailed below). These conflicts are translated into ‘instability years’ as explained above, and then aggregated into 10-year bins for analysis.

### Endogenous factors: PSI

We follow here the methods employed in previous studies investigating periods of societal instability utilizing Structural Demographic Theory (SDT). These studies quantified societal pressure as a single composite measure–the Political Stress Index (PSI)–made up of three primary components: Mass Mobilization Potential (MMP), Elite Mobilization Potential (EMP), and State Fiscal Distress (SFD). For the present article, these components were calculated as follows:

**MMP** is measured as the inverse of arable land per capita. This was measured by dividing estimates of the yearly population during the Qing period(adapted from the ClioInfra project; data accessed August 2022) by estimates of the total amount of arable land available for agricultural production by year [58–62]. The inverse of this ratio is used to capture the expectation that a *decrease* in the availability of arable land per capita will add additional pressure on popular well-being, thus raising PSI.

Certainly other measures were possible. Notably, data on price levels for staple goods like rice or on labourers real wages [63] (namely, average wages adjusted for the price of key goods, so incorporate price levels) are likewise good indicators of popular well-being that have been utilized in previous SDT works [53]. We chose to focus on arable land, as the majority of the Qing population were employed in agricultural production at this time, rather than wage labour. Thus, we expect that the availability of productive land would have the strongest impact on popular well-being in this period. Note though that the available estimates of how real wages changed during this period correspond very closely to the dynamics of per-capita land availability: the correlation coefficient is 0.77; see also Fig 2 illustrating the similar dynamics of both measures. Using real wages rather than land availability does not make a substantial difference in the results, nor does utilizing a composite measure.

**EMP** is measured as the inverse of the number of *jinshi* degrees awarded yearly per capita [84–87]. As noted in the main text, the *jinshi* were the highest degree holders. Unfortunately we lack clear, reliable estimates of the elite population or yearly estimates of the number of applicants for the *jinshi* degree specifically. Based on findings from previous work, however, we estimate that the number of potential applicants would scale with population, so a shrinking ratio of degrees granted per total population serves here as a leading indicator of mounting competition for these prestigious degrees. We explain in the main text that other indicators from the Qing period bear this out. The inverse is used as *falling* rates of *jinshi* degree-holders per capita should lead to an increase in elite competition, also raising PSI.

**SFD** is measured as the deficit faced by state fiscal resources. We first derive the Qing’s fiscal balance by subtracting estimated yearly revenue from estimated yearly expenditures [62]. Revenue here accounts for recorded incomes in the form of taxes (as silver-equivalent), whether paid in coin, kind, or labour. Expenditures include costs paid by state coffers, excluding extra revenues as from merchant contributions to war efforts. We then divided the balance by revenue to produce a running ratio of how much revenues exceeded expenses, using the inverse of this figure as our measure of state surplus/deficit. The inverse is used to indicate that when revenues exceeded expenses, there was no fiscal distress (these values are negative) and therefore we expect SFD and thus PSI to decrease, but when there was a deficit then fiscal distress was present (so these numbers are positive), increasing PSI.

While the Qing period is generally well documented, we lack reliable estimates for every datapoint for every year for all of these different variables. Note that in the main text, we provide information for the years available, even though the values are not always reported for the same year in each case (for instance, reliable population figures and estimates of available arable land, or candidates for *jinshi* exams). To estimate the missing data, we employed LOESS interpolations, resolved at a decadal scale.

Overall, this produces time-series data extending for the entire Qing period, with an estimate every 10 years between 1640 and 1910 for each of the measures serving to proxy the three primary components of SDT, MMP, EMP, and SFD. **PSI** is then constructed by multiplying these three measures together. This gives a total of 28 decadal estimates between 1640 and 1910 for each of the factors described here.

### Exogenous factors

#### Ecological shocks

As we describe in the main text, several scholars in recent years have pointed to ecological shocks as being major, or even the most significant, driver of the Qing collapse [108–115]. Here, we focus on the two types of disasters raised as the most devastating for popular well-being and generating long-lasting social instability: major droughts and famines. We adopt data from the REACHES dataset [45] (Data accessed August 2022), a large time-series of ecological disasters experienced in China during Qing rule. The dataset combines historical and documentary evidence with ecological evidence such as pollen traces and tree-ring data. The dataset lists the number of sites experiencing different types of ecological disasters annually.

#### External warfare

Data on wars fought by the Qing against foreign states are taken from the same sources and following the same methodology as Internal War.

## Regression analysis

We performed linear regression analysis to determine the relationship between our PSI measure and our primary measure of social instability, Internal War (described above). This provided us with 140 datapoints (28 observations at decadal intervals across 5 factors: PSI, Extrenal War, Droughts, Famines, and Internal War).

First, we find that PSI displays a significant positive relationship with Internal War: p-value = 1.85 × 10^−5^; adjusted R^2^ = 0.5. Further, exploring all combinations of the individual measures that compose PSI–MMP, EMP, SFD–produces weaker fits (by R^2^) than the combined PSI measure alone (S1 Table in S1 File).

Next, we explored the relationship between internal conflict and exogenous factors–ecological disasters and external warfare (as well as PSI). The regression did not produce significant relationships except PSI (S2 Table in S1 File).

Finally, we explored the causal relationship between these factors using a dynamic regression technique [130] (S3 Table in S1 File). We assess whether a change in the different factors at one time-step would be significantly related to a change in internal conflicts in the next time-step. Here a time-step is 10 years; thus, we fit a regression model where the response (dependent) variable is our measure of internal conflict at year *t* against PSI, Droughts, Famines, and External War at year *t*-1, namely 10 years earlier. This sort of ‘Granger causality’ has been employed to determine causal connections between theoretically-implicated factors using time-series historical data similar to what we have collected for the Qing period [131,132]. We include also internal conflict at time *t*-1 to assess the strength of autocorrelation; in other words, we want to see how much the number of internal conflicts being waged under the Qing in one decade can be predicted by the number of conflicts a decade earlier, which would indicate that there was a conflicts were long-lasting or somewhat self-perpetuating.

We find with this dynamic regression that PSI along with the autocorrelation term (Internal War*_t-1_*) are the only factors that display a significant relationship with internal conflicts in the next time-step, with PSI being the strongest (by t-value). Neither Droughts and Famines nor External War display a significant relationship with internal conflicts, even when using this dynamic regression approach. Indeed, this model, with the time-lagged terms, offers the most explanatory power compared with the ‘synchronous’ regression as indicated by the R^2^ term (S2 Table in S1 File).

Overall, these results offer strong support to the conclusions expressed in the main text that rising social pressures, proxied here by PSI, are a better predictor for the instability experienced by the Qing than ecological disaster. Further, this supports the conclusion that PSI is a leading indicator, rising first before the pressure materializes as realized conflicts.

## Discussion

Our analysis suggests that SDT provides a compelling and comprehensive framework for understanding how rising sociopolitical pressures, peaking in the mid-19^th^ century, resulted in a series of rebellions and insurrections, which killed tens of millions of Chinese during the Qing Dynasty. Ultimately, population growth was a leading driver of rising social pressures throughout the Qing reign; China witnessed a massive, four-fold population increase between 1700 and 1840, which greatly reduced arable land per capita and led to the impoverishment of the rural population. Declining wages and decreasing statures in the eighteenth century provide additional signals of a population under severe strain.

Elite overproduction followed as the second major driver of instability. While the elites during the Qing dynasty included both landholders with an imperial degree and nonofficial gentries (landholders with no imperial degree), degree-holders and especially literati still constituted the most important portion of the Qing elites, as they possessed greater ability to exert influence over the government and the non-elite population. The Qing’s inelastic institutional system, mostly inherited from the Ming (when the population was substantially smaller), was unable to adequately absorb the burgeoning numbers of aspirant elites who had invested in expensive educations in order to increase their chances of obtaining a position in the government bureaucracy. However, as the numbers of aspirants increased several-fold, the number of the highest degrees awarded decreased, reaching its lowest point in 1796. This increased the frustration and hostility of those aspirants who repeatedly failed the examinations. It is emblematic that the Taiping Rebellion was launched and coordinated by a group of Hakka leaders who had failed the examinations. The Taiping leaders were clearly capable organizers, since they had acquired the ideological and technical know-how to coordinate revolts and mobilize the impoverished and equally frustrated non-elite population. Indeed, peasant farmers, miners, and charcoal burners made up a significant portion of the rebel forces. Towards the end of the dynasty, elite overproduction showed itself in the masses of educated elites who argued for major reform of the political system, including the end of the empire and the establishment of a republic. The Petition of the Examination Candidates (*Gongche shangshu*) of 1895 and the Hundred Days’ Reform movement of 1898 are two representative examples of political movements led by educated reformists, such as Kang Youwei (1858–1927), Liang Qichao (1873–1929), and Tan Sitong (1865–98), who asked for the establishment of a modern educational and institutional system.

Finally, Qing state capacity began to break down as well by the mid-19^th^ century. The growing costs of suppressing unrest, declining per capita productivity, successive financial shocks and growing trade deficits driven largely by dwindling silver supplies and opium imports, and increasing pressure from external foes such as the English, French, and Japanese placed the Qing state under severe strain, compromising their ability to meet challenges that had been managed effectively or at least quickly recovered from earlier in the reign. Although the Qing did manage to hold out for several decades in the face of high PSI, eventually these mounting forces proved too much to handle, leading to the Qing collapse in 1911.

It would be a mistake to conclude that the Qing government was unaware of the severity of these pressures. At the beginning of the dynasty, institutional reforms, such as state granary and other famine relief policies, contributed to restoring popular well-being and neutralizing some of the major drivers that had precipitated the fall of the Ming. By the mid-19^th^ century there was a fairly elaborate famine-relief system [133], substantial state granaries, local markets were mushrooming in the lower Yangzi delta region and Jiangnan, and early investments in industrialization improved mining enterprises in regions such as Yunnan. The number of schools and academies also increased significantly, in order to accommodate the new aspirant elites who had obtained at least the degree of *shengyuan* within the power structure, and fixed quotas for ethnic minorities were implemented to promote equality and fairness. Nevertheless, these measures proved unable to hold off rising pressure, particularly as state capacities were stretched beyond their limits as the 19^th^ century unfolded and the systems put in place to ameliorate suffering among the population broke down.

Previous work attempting to diagnose the causes that led to the Qing collapse have stressed the importance of ecological shocks, of economic decline, of external conflict, or of ethnic divides and ideological motivations for revolt against an imperial power. Our analyses suggest that all of these factors played a role, but none are able *by themselves* to account for the complete picture. The SDT framework provides a structure to explain how these factors not only grew in response to population pressure and elite overproduction, but interacted with each other. Our measure of these combined social pressures, PSI, peaked between 1840 and 1890, a period of the largest rebellions of the late Qing era. The survival of the dynasty during this period is another indication of the great resilience of the Qing institutional structures in the face of enormous internal and external shocks that came one after another during the nineteenth century. The Qing government not only managed to suppress the rebellions of 1850s and 1860s, but during the late 19^th^ century these pressures somewhat abated (Fig 4). The MMP decline was primarily a result of population decrease, caused by internal warfare, but EMP was relieved by creating additional positions in the bureaucracy. At the same time the Qing court did try to create a vast array of state-sponsored industrial enterprises. However, the Qing’s industrialization efforts were hampered, and eventually defeated by the rise of the increasingly powerful military elites, who siphoned off, for their personal advantage, significant portions of the surpluses that had not been destroyed by the rebellions [134]. The emergence of the military elite, and the waning power of the administrative ones, led to the spread of warlords and factionalism among leaders who were previously instrumental in the Qing’s military successes [135]. These trends plagued the last decades of the Qing Dynasty and the Republican period. Finally, although the Qing were attempting to address these internal pressures during this final period, the arrival of new powerful external geopolitical challengers proved one challenge too many for the dynasty.

Seen from this perspective, the Qing dynasty followed a path to collapse similar to that of another powerful Eurasian empire, governed by the nearly contemporaneous Romanov Dynasty (1613–1917) [136]. Between 1613 and 1860 the population of Russia increased from less than 5 million to more than 60 million. Although the Russian Empire had extended its territory, such a rapid population growth far exceeded the stocks of arable land available to peasants, leading to their immiseration and increased mass-mobilization potential. The decline in the average height of Russian peasants by 4 cm during the 18^th^ century parallels the similar decline in China. At the same time, the elite numbers also increased, as did their consumption levels. The combination of elite overproduction and decreased state legitimacy following the humiliating defeat of the Tsarist regime in the Crimean War (1853–56) led to the creation of a huge number of frustrated elites, a development that in turn fed the growth of radical organizations, such as anarchist and social revolutionary movements.

Although Russia during the 1850s and 1860s experienced a wave of peasant agitation and protests, and a terrorist campaign that ultimately claimed the Tsar as its victim, unlike China, during the 19^th^ century Russia avoided a major civil war, along the lines of the Taiping Rebellion, or a revolution, similar to those that swept Europe in 1848–49. Instead, during the 1860s the Romanov government adopted the Great Reforms that proved to be successful in reducing social tensions at least for a while. The Romanov Dynasty even survived the Revolution of 1905–07, only to be brought down at the end of World War I, which proved to be a geopolitical challenge that the dynasty failed to surmount. As a result, both the Qing and Romanov dynasties were destroyed by a combination of elite overproduction, the exposure to new radicalizing social ideologies such as Marxism, and the pressure from external geopolitical forces.

Although beyond the scope of the present paper, we believe our structural-demographic analysis of the Qing demonstrates that the consistent analysis of China’s millennia-old imperial history using this framework provides insight about the causal mechanisms driving the much-discussed empirical pattern of the cyclical rise-and-fall of China’s many dynasties, starting with the early Qin and Han empires in the 3rd century BCE [44]. These future analyses, using a common causal framework, could provide the comparative potential to better understand not only other cases of past state breakdown, but of current and future crises as well. In fact, many contemporary societies around the world are experiencing a suite of pressures very similar to those that faced the Qing. SDT has been utilized not only to explain the instability experienced by these more modern societies, but to forecast social pressure before it reaches the ‘boiling point’ [31,137,138]. Admittedly, the time scales of these growing instabilities are long and the sort of deep structural forces we highlight here typically do not form part of normal political discourse. We believe, however, that insights from past crises, suitably analyzed, have the potential to inform present and future policy, potentially enabling societies and leaders to navigate such crises through peaceful reform.

## Supporting information

S1 File Supporting Information Tables. File including supporting information tables S1 to S3 Tables.
(DOCX)
