# Supplementary material for: Structural-demographic analysis of the Qing Dynasty (1644–1912) collapse in China
Source: PLoS One. 2023 Aug 18;18(8):e0289748. doi: 10.1371/journal.pone.0289748 (PMC10437944; doi:10.1371/journal.pone.0289748)
Supplement: S1 File — File including supporting information tables S1 to S3 Tables. (DOCX) [file pone.0289748.s001.docx]

# Supporting Information

**Structural-Demographic Analysis of the Qing Dynasty (1644–1912) Collapse in China**

Georg Orlandi, Daniel Hoyer, Hongjun Zhao, James S. Bennett, Majid Benam, Kathryn Kohn, Peter Turchin

| **Model (# variables)** | **PSI** | **MMP** | **EMP** | **SFD** | **R^2^** |
| --- | --- | --- | --- | --- | --- |
| model 1 ( 1 ) | * |  |  |  | 0.4936281 |
| model 2 ( 2 ) | * |  |  | * | 0.4922951 |
| model 3 ( 2 ) | * |  | * |  | 0.49147698 |
| model 4 ( 3 ) | * | * | * |  | 0.48554001 |
| model 5 ( 2 ) | * | * |  |  | 0.47727779 |
| model 6 ( 3 ) | * |  | * | * | 0.4751726 |
| model 7 ( 3 ) | * | * |  | * | 0.47114251 |
| model 8 ( 4 ) | * | * | * | * | 0.4648926 |
| model 9 ( 2 ) |  |  | * | * | 0.42934233 |
| model 10 ( 3 ) |  | * | * | * | 0.41279252 |
| model 11 ( 2 ) |  | * |  | * | 0.3925865 |
| model 12 ( 1 ) |  |  |  | * | 0.3305048 |
| model 13 ( 1 ) |  | * |  |  | 0.27767274 |
| model 14 ( 2 ) |  | * | * |  | 0.25295758 |
| model 15 ( 1 ) |  |  | * |  | 0.18355701 |

**S1 Tab. Summary of Regression Results: Examining Relationships between Internal War and PSI, Exogenous Factors, and Causality.** Results of exhaustive search regression, exploring relationship of every combination of factors with Internal War. Asterisks show which factors were included in each model variant; rows are sorted by R^2^, with highest (best performing) models on top.

|  | **Coefficient** | **t-value** | **Significance**  **(p-value)** |
| --- | --- | --- | --- |
| (Intercept) | 1.21 | 0.27 | 0.79 |
| PSI | 30.88 | 4.76 | 8.39*x*10^-05^ *** |
| Drought | 0.001 | 0.09 | 0.927 |
| Famine | 0.003 | 0.19 | 0.85 |
| External War | 0.1 | 0.22 | 0.64 |

**S2 Tab. Results of regression model of Internal War against exogenous factors**

*Overall Adjusted R^2^ = 0.45*

**** = significance at > 0.001 threshold*

** = significance at 0.01 threshold*

*^+^ = significance at 0.05 threshold*

|  | **Coefficient** | **t-value** | **Significance**  **(p-value)** |
| --- | --- | --- | --- |
| (Intercept) | 1.46 | 0.33 | 0.74 |
| Internal War*_t-1_* | 0.36 | 1.9 | 0.07^+^ |
| PSI*_t-1_* | 20.9 | 2.4 | 0.02* |
| Drought*_t-1_* | -0.00008 | -0.007 | 0.99 |
| Famine*_t-1_* | 0.004 | 0.3 | 0.77 |
| External War *_t-1_* | -0.02 | -0.1 | 0.92 |

**S3 Tab. Results of dynamic regression of Internal War against factors time-lagged by 10 years**

*Overall Adjusted R^2^ = 0.54*

**** = significance at > 0.001 threshold*

** = significance at 0.01 threshold*

*^+^ = significance at 0.05 threshold*
